# Brain Metastasis in Papillary Serous Adenocarcinoma of the Endometrium

**DOI:** 10.1055/s-0039-1683353

**Published:** 2019-04-16

**Authors:** Walberto Monteiro Neiva Eulálio Filho, Taíla Sousa de Moura Fé, Rodolfo Myronn de Melo Rodrigues, Maria Simone Oliveira Lima, Sabas Carlos Vieira

**Affiliations:** 1 Department of Specialized Medicine, Universidade Federal do Piauí, Teresina, PI, Brazil; 2 Centro Universitário Uninovafapi, Teresina, PI, Brazil

**Keywords:** endometrium, serous adenocarcinoma, metastasis, brain, endométrio, adenocacinoma seroso, metástase, cérebro

## Abstract

**Background** Most endometrial cancers (75%) are diagnosed in early stages (stages I and II), in which abnormal uterine bleeding is the most frequent clinical sign. When the diagnosis is performed in stage IV, the most common sites of metastasis are the lungs, liver and bones. Central nervous system (CNS) metastasis is a rare condition. The aim of this study is to describe a case of uterine papillary serous adenocarcinoma of the endometrium that progressed to brain and bone metastases.

**Case Report** We present the case of a 56-year-old woman with abnormal uterine bleeding and endometrial thickened echo (1.8 cm). A hysteroscopy with biopsy was performed, which identified poor differentiated serous adenocarcinoma of the endometrium. A total abdominal hysterectomy, with pelvic and para-aortic lymphadenectomy, was performed. Analysis of the surgical specimen revealed a grade III uterine papillary serous adenocarcinoma. Adjuvant radio/chemotherapy (carboplatin and paclitaxel—six cycles) was indicated. Sixteen months after the surgery, the patient began to complain of headaches. Brain magnetic resonance imaging demonstrated an expansile mass in the right parietal lobe, suggesting a secondary hematogenous implant subsequently confirmed by biopsy. She underwent surgery for treatment of brain metastasis, followed by radiotherapy. She died 12 months after the brain metastasis diagnosis due to disease progression.

**Conclusion** Uterine papillary serous adenocarcinoma of the endometrium has a low propensity to metastasize to the brain. To the best of our knowledge, this is the fifth documented case of uterine papillary serous adenocarcinoma of the endometrium with metastasis to the CNS.

## Abstract (pt)

**Resumo:**

**Fundamentos** A maioria dos cânceres de endométrio (75%) é diagnosticada em estágios iniciais (estágios I e II), nos quais o sangramento uterino anormal é o sinal clínico mais frequente. Quando o diagnóstico é realizado no estágio IV, os locais mais comuns de metástase são os pulmões, o fígado e os ossos. A metástase para o sistema nervoso central (SNC) é uma condição rara. O objetivo deste estudo é descrever um caso de adenocarcinoma seroso-papilífero do endométrio que progrediu para metástases cerebral e óssea.

**Relato de Caso** Apresentamos o caso de uma mulher de 56 anos com sangramento uterino anormal e eco endometrial espessado (1,8 cm). Foi realizada histeroscopia com biópsia que identificou adenocarcinoma seroso-papilífero pouco diferenciado do endométrio. Uma histerectomia abdominal total, com linfadenectomia pélvica e para-aórtica, foi realizada. A análise da peça cirúrgica revelou adenocarcinoma seroso-papilífero do endométrio grau III. Radioterapia adjuvante/quimioterapia (carboplatina e paclitaxel—seis ciclos) foi indicada. Dezesseis meses após a cirurgia, a paciente começou a se queixar de dores de cabeça. A ressonância magnética cerebral demonstrou uma massa expansiva no lobo parietal direito, sugerindo um implante hematogênico secundário posteriormente confirmado por biópsia. A paciente foi submetida a cirurgia para tratamento de metástase cerebral, seguida de radioterapia. A paciente morreu 12 meses após o diagnóstico de metástase cerebral devido à progressão da doença.

**Conclusão** O adenocarcinoma seroso-papilífero do endométrio tem uma baixa propensão a metastizar para o cérebro. Até onde sabemos, este é o quinto caso documentado de adenocacinoma seroso-papilífero do endométrio com metástase para o SNC.

## Introduction

Endometrial cancer is the seventh most common malignancy worldwide. Up to 75% of endometrial neoplasms are diagnosticated as early-stage disease (stages I and II), because the main and early symptom is abnormal uterine bleeding, which occurs in 90% of women, especially in the postmenopausal period. The investigation includes transvaginal ultrasonography (TVU) and hysteroscopy with biopsy.^1^ ^2^

There are several risk factors related to endometrial cancer. They are mainly involved in prolonged exposure to endogenous estrogens, such as obesity, early age at menarche, nulliparity, late-onset menopause, older age (≥ 55 years), and use of tamoxifen. The relation between diabetes and endometrial cancer is controversial. It appears that obesity usually combined with diabetes mellitus type 2 may be the real cause of the higher incidence of this type of cancer in this population. On the other hand, the levonorgestrel-releasing intrauterine system might have a protective effect against endometrial malignant transformation.^3^

Its pathogenesis involves two distinct processes. Type I or endometrioid is the most frequent and consists of a low-grade carcinoma that evolves from endometrial hyperplasia and presents a good prognosis (overall survival 85% at 5 years). Its origin involves, in 30 to 40% of cases, loss of DNA mismatch repair proteins through mutations in the genes MLH1, MSH2, MSH6, and PMS2. Type II, or non-endometrioid, includes serous, carcinosarcoma and clear-cell subtypes. It is much more aggressive, presenting 55% survival in 5 years, and its origin is mainly related to mutation in the P53 gene.^3^ Among the various subtypes of endometrial cancer, papillary serous adenocarcinoma is very uncommon, accounting for only 6.5% of all endometrial tumors.^4^

The main pathways of dissemination of endometrial carcinoma are direct invasion of the myometrium and uterine serosa or lymphatic dissemination to pelvic and para-aortic lymph nodes. Intra-abdominal metastasis usually results from the passage of malignant cells through the fallopian tubes. Major sites of hematogenous spread are the lungs, liver and bones. Brain metastasis is extremely rare.^5^ The aim of this study is to describe a case of papillary serous adenocarcinoma of the endometrium that progressed to brain and bone metastases.

## Case Report

**Fig. 1:**
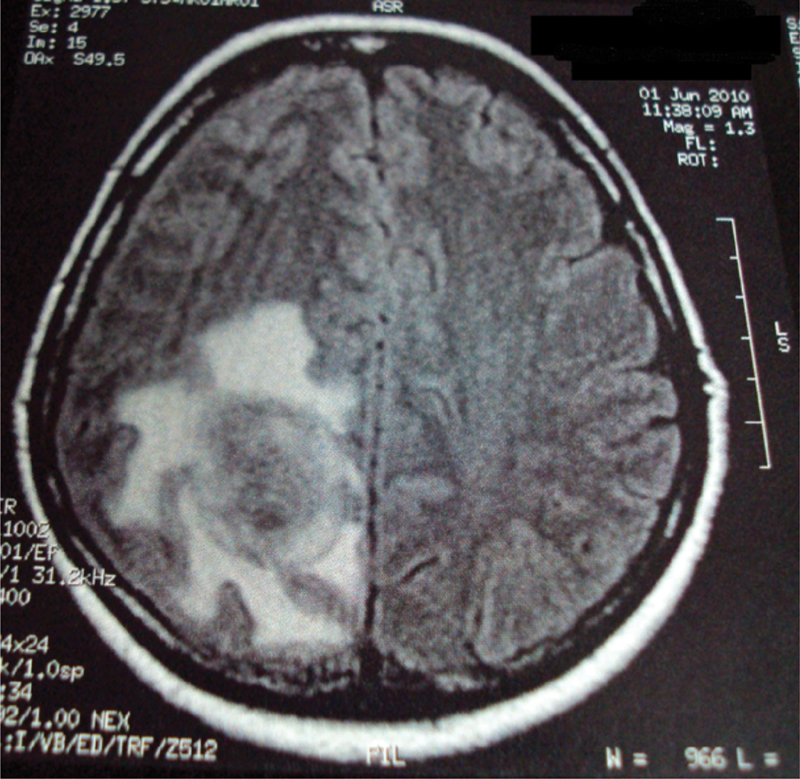
Magnetic resonance imaging showing an expansile mass in the right parietal lobe consistent with hematogenous implant.

A 56-year-old, G2P2A0, menopausal, married patient sought gynecologic care with a history of abnormal uterine bleeding, initiated in August 2008. Her individual and gynecological history were unremarkable, except for controlled hypertension. She denied diabetes, smoking and alcoholism. The pelvis and abdomen physical examination was unremarkable. Gynecological examination revealed the following: a cervix with no visible abnormalities, physiological secretion, no signs of infections, and a normal uterine bimanual digital examination. Mammography and ultrasonography had no alterations. Transvaginal pelvic ultrasonography demonstrated a thickened endometrial echo (18 mm), with signs of hypervascularization on Doppler scan. In January 2009, a hysteroscopy with endometrial biopsy was performed and revealed a poor differentiated serous adenocarcinoma of the endometrium. A total abdominal hysterectomy, with pelvic and para-aortic lymphadenectomy, was performed. During exploration of the abdominal cavity, no signs of local or distant carcinomatosis were identified. The patient underwent bilateral adnexectomy. Since the greater omentum was macroscopically normal, supracolic omentectomy was not performed. Analysis of the surgical specimen revealed a 2 cm grade III uterine papillary serous adenocarcinoma of the endometrium with lymph-vascular space invasion. Surgical margins and six lymph nodes were free of disease. Cytology of peritoneal fluid resulted negative. The pathological analysis could not conclude a complete clinical stage according to the FIGO (International Federation of Gynecology and Obstetrics) classification. Initially, the patient's FIGO classification was considered IB G3. After review of criteria and inclusion of omentectomy for clinical stage, it was not possible to define between stage IB and III A because the omentectomy was not performed. Adjuvant radio/chemotherapy was indicated. External radiotherapy was performed (5,000 cGy in 25 fractions) during the month of May 2009, followed by 6 cycles of chemotherapy with carboplatin and paclitaxel, which was completed in October 2009. On day 1 of each chemotherapy cycle, the patient received paclitaxel, 175 mg/m^2^, intravenously (IV) over 3 hours, carboplatin area under the curve (AUC) 6 IV over 30 minutes, and the treatment cycle was repeated every 3 weeks. The patient received follow-up care and remained without complaints until April 2010, when she reported episodes of headache and visual disturbance. A brain magnetic resonance imaging (MRI) was performed and identified an expansile mass in the right parietal lobe (Fig. 1), suggesting a secondary hematogenous implant.

The level of tumor marker CA-125 found was 11.4 U/mL (reference value ≤ 35). The patient underwent a craniotomy for tumor excision to relieve compression of the adjacent structures. The procedure occurred without complications, with complete excision of the lesion. Histopathologic examination revealed metastasis of a uterine papillary serous adenocarcinoma of the endometrium with free margins. External radiotherapy (4,000 CGY in 20 fractions) was then performed in July 2010. At follow-up, a new brain MRI in October 2010 showed a lobulated mass in the right parieto-occipital region. In January 2011, a new craniotomy with complete metastasis excision and tumor-free margins was performed due to return of the symptoms, followed by a new cycle of radiotherapy (4,000 CGY in 20 fractions). The patient complained of severe pain in the lower dorsal region in March 2011. At this time, the bone scintigraphy demonstrated secondary neoplastic lesions in the cranial vault, distal half of the femurs and proximal end of the tibias. The patient was placed in palliative care with the use of opioids for pain management and psychological counseling. She died on May 2011 due to the progression of the disease.

## Discussion

The endometrial cancer survival depends on multiple factors, such as tumor grade, age, comorbidities, tumor diameter, American Society of Anesthesiologists score, lymphovascular space involvement, and postoperative complications at 30 days. Currently, the best predictor of survival is the FIGO stage. A total of 75% of the cases are diagnosed at an early FIGO stage (I and II), whose 5-year overall survival ranges from 74 to 91% in patients without lymph node metastasis, 60 to 70% in those with pelvic lymph node metastasis, and 30 to 40% in those with para-aortic lymph node metastasis. The 5-year overall survival rate for FIGO stage III is 57 to 66%; for FIGO stage IV, it is 20 to 26%.^3^

Papillary serous adenocarcinoma is uncommon among the diverse histologic types of endometrial cancers, accounting for 6.5% of all endometrial tumors.^4^ Endometrial carcinoma has a pattern of distant metastasis preferentially to the lungs and liver. Metastasis to the central nervous system (CNS) is rare. The incidence of brain metastasis ranges from between 0.3 and 1.4% in clinical settings to between 1 and 3% in autopsy series among the various subtypes of endometrial cancer.^6^ Slightly more than 100 cases of CNS implants were documented. When CNS implants occur, the disease is usually at an advanced stage and has disseminated to multiple organs.^6^ ^7^ After extensive literature review, only 4 cases of uterine papillary serous adenocarcinoma with metastasis to the CNS have been reported, which justifies this case presentation.^8^ ^9^ ^10^

Signs and symptoms of this type of metastasis resemble those of other CNS metastases, such as mental confusion (45%), gait disturbance (40%), paralysis (20%), dysarthria (10%) and nausea or vomiting (10%).^11^ Diagnosis is established by computed tomography and MRI in 40% of the cases; tomography alone in 20%, and MRI alone in 40%. Data suggest that in the presence of neurologic symptoms in a patient with endometrial cancer, the investigation of brain metastasis should include MRI as the first-line imaging modality.^11^

In patients with brain metastasis, death occurs at around 7 weeks after the appearance of symptomatic lesions if left untreated. Lesions generally progress with increased intracranial pressure and consequent brain herniation with brainstem compression. Corticoids are the mainstay of treatment of cerebral edema. In patients with a solitary metastasis, surgery is an important therapeutic option that allows local tumor control and significantly improves focal deficits caused by mass effect.^12^

The current recommendation for the treatment of a solitary brain metastasis in a patient in good general health is surgical resection or radiosurgery followed by whole brain radiation. Multimodal treatment of brain metastasis (surgery + radiotherapy + / - chemotherapy) is beneficial to overall survival (median survival of 9.2 months), in comparison to radiotherapy alone (median survival of 0.9 months) (*p* = 0.0001) or therapeutic abstention (median survival of 0.2 months) (*p* = 0.009).^13^ In the case in question, the patient initially had a solitary metastasis, and surgical removal was indicated, with radiotherapy supplementation for improvement of symptoms. After the appearance of bone metastasis in multiple sites and rapid deterioration of the clinical condition of the patient, palliative care was chosen.

## Conclusion

Uterine papillary serous adenocarcinoma of the endometrium has a low propensity to metastasize to the brain. To the best of our knowledge, this is the fifth documented case, to date, of uterine papillary serous adenocarcinoma of the endometrium with metastasis to the CNS. This case corroborates data available in the literature portraying brain metastasis as a poor prognostic sign with a low survival rate.
